# Costs associated with anastomotic leak after left-sided colorectal surgery: retrospective real-world study in England

**DOI:** 10.1093/bjsopen/zrag049

**Published:** 2026-05-20

**Authors:** Julia Glover-Kirtland, Cindy Tong, Melek Pinar Bosut, Niels-Derrek Schmitz, Sara Joao Carvalho, Caoimhe T Rice, Dion G Morton, Elizabeth Li, Thomas D Pinkney

**Affiliations:** Health Economics and Market Access, Johnson & Johnson MedTech, Berkshire, UK; Health Economics and Market Access, Johnson & Johnson MedTech, New Brunswick, New Jersey, USA; Health Economics and Market Access, Johnson & Johnson Medical GmbH, Norderstedt, Germany; Health Economics and Market Access, Johnson & Johnson Medical GmbH, Norderstedt, Germany; Thermo Fisher Scientific, London, UK; Thermo Fisher Scientific, London, UK; Academic Department of Surgery, University of Birmingham, Birmingham, UK; Academic Department of Surgery, University of Birmingham, Birmingham, UK; Academic Department of Surgery, University of Birmingham, Birmingham, UK

**Keywords:** clinical outcomes, healthcare costs, hospitalizations, length of hospital stay, matched cohort, postoperative complication

## Abstract

**Background:**

Anastomotic leak (AL) is a serious postoperative complication following colorectal surgery (CS). This study compared healthcare resource utilization (HCRU) and costs between patients with and without AL after left-sided CS to highlight the significant burden of AL, and to address the evidence gap regarding its economic impact in England.

**Methods:**

This was a retrospective matched cohort study of patients with and without AL after left-sided CS. Data for adult patients undergoing CS between 2018 and 2021 in England were drawn from the Hospital Episode Statistics database. Patients with and without AL after left-sided CS were exactly matched 1 : 1 on age, sex, surgery date and type, and indication. Comparative analyses were conducted on patients with *versus* without AL, as well as on patients with minor and major AL *versus* those without AL. The primary objective was to compare all-cause costs for hospitalization; secondary outcomes were to determine AL incidence rates, baseline characteristics of those with AL (major and minor) *versus* those without, and the implications of AL in terms of interventions and length of hospital stay (LOS). Multivariate models were adjusted for the Charlson co-morbidity index and geographical region.

**Results:**

Of 36 948 adult patients who underwent left-sided CS with anastomosis without stoma formation, after excluding those with bleeding complications, 2059 patients with AL and 27 590 without AL were identified. After exact matching, there were 1982 patients in each group. Among patients with AL, 1116 had major AL and 866 had minor AL. AL was associated with a significantly higher mean all-cause inpatient cost (adjusted difference €11 728; 95% confidence interval (c.i.) €10 313 to €13 209; *P* < 0.001). Patients with AL also had a significantly higher mean cumulative LOS (adjusted difference 15.93 days; 95% c.i. 14.24 to 17.61 days; *P* < 0.001). The estimated incidence of AL requiring intervention was 5.6%. Patients with any AL had significantly more mean all-cause hospitalizations (adjusted difference 0.37; 95% c.i. 0.26 to 0.49; *P* < 0.001) and higher cumulative LOS (adjusted difference 15.93; 95% c.i. 14.24 to 17.61; *P* < 0.001) than patients without AL.

**Conclusion:**

AL is associated with poorer clinical outcomes, increased HCRU, and a substantial economic burden.

## Teaser

This retrospective matched cohort study compared healthcare resource utilization and costs between patients with and without anastomotic leak (AL) after left-sided colorectal surgery to highlight the significant burden of AL and to address the evidence gap regarding its economic impact in England. AL was associated with a significantly higher mean all-cause inpatient cost of €11 723 and a significantly higher mean cumulative length of hospital stay of 15.93 days.

## Introduction

Anastomotic leak (AL) is one of the most serious postoperative complications after colorectal surgery, which, despite surgical and technological advances, remains a significant challenge for patients and health services^1^. Currently a standardized definition of AL is lacking^2^. The International Study Group of Rectal Cancer (ISREC) proposed defining AL as a defect of the intestinal wall integrity at the colorectal or coloanal anastomotic site, leading to a communication between the intra- and extraluminal compartments^3^. Subsequent studies have been published on the definition and management of ALs^4–6^. The reported incidence of AL varies from 1.5 to 23%^7^, and is modified by patient selection, surgeon, and operative environment, as well as the surgical site and techniques used. In a global study from 49 countries published by the European Society of Coloproctology (ESCP) in 2018, the 30-day postoperative AL rate was 8.6% in left-colon, sigmoid, and rectal resections^8^. AL is associated with poor clinical outcomes and increased short- and long-term morbidity and mortality^9–14^. Patients who develop AL have a higher risk of postsurgical complications, including multiple organ failure and sepsis, than those without AL^13,14^. Patients with AL require greater postoperative care, which may include intensive care, extended hospital stays, and the possibility of reoperation, along with higher readmission rates^11,15,16^. This, in turn, leads to increased healthcare resource utilization (HCRU) and healthcare costs^3,15–20^. Studies from different countries have estimated that, compared with patients not developing AL, total inpatient costs increase by 39.9 (Romania) to 513.1% (Brazil) with AL^3,18,19,21,22^. A 2013 publication reported that, in England, an underestimation of the true cost of AL following anterior resection may result in an estimated shortfall of up to £2.4 million (€2.64 million, based on 2013 purchasing power parity^23^) per year to secondary care providers^18^, with the greatest overall driver of cost being the length of hospital stay (LOS)^16,17^. Hospital readmissions and the need for intensive care and reinterventions have been identified as key factors in increased healthcare costs^21^. AL is also associated with significant environmental impact, driven by resource usage both in hospital and during home care^24^.

The primary aim of the present study was to compare all-cause cost for hospitalization between patients with and without AL after left-sided colorectal surgery to highlight the significant burden of AL and the importance of minimizing the risk in clinical practice. Secondary aims were to determine AL incidence rates and the baseline characteristics of those with AL (both major and minor) *versus* those without. This research addressed this evidence gap by using matched-cohort cost analyses from real-world National Health Service (NHS) data to determine the economic impact of AL in England.

## Methods

### Study design

This was a retrospective real-world matched cohort study to quantify the incidence and economic impact of AL requiring intervention after left-sided colorectal surgery in the English NHS. Patients were identified from the Hospital Episode Statistics (HES) database produced by NHS Digital. The HES database contains details of all admissions, emergency care attendances, and outpatient appointments at NHS hospitals in England^25^. All adult patients in England aged ≥ 18 years (on the date of surgery) who had left-side colorectal surgery with creation of an anastomosis between 1 January 2018 and 31 December 2021 (identification window) were identified from the HES data (*Fig. S1*). An additional baseline period from 1 April 2017 until the index surgery, defined as the date of first colorectal surgery, was used to determine patient characteristics, and to exclude those with previous colorectal surgery.

### Study participants

Adult patients who had left-sided colorectal surgery (both for malignant and benign disease) with the creation of an anastomosis during the identification window were included in the study. AL was defined as patients requiring further surgical or non-surgical intervention(s) between 1 and 30 calendar days after the first colorectal surgery during the identification window (index surgery), identified using procedure codes and diagnosis codes (*Table S1*).

The coding schema for the identification of ALs was developed based on ISREC grade B and C, insofar as possible, within the limits of the data set. It should be noted that no laboratory test results or radiological reports are available from the HES data set; thus, the coding schema focused on additional procedures. The reoperation procedure codes were used to identify major AL (equivalent to ISREC grade C) and siting of additional drains, or a diagnosis of abscess or peritonitis without reoperation for minor AL (equivalent to ISREC grade B). It was not possible to identify patients with ISREC grade A ALs because these do not require intervention.

Non-surgical interventions, including total parenteral nutrition, image-guided drainage, the management of abscess, infection, and/or acute peritonitis were considered indicative of minor AL (*Table S2*). For the purposes of this study, the need for reoperation, washout procedure, reanastomosis, colostomy, laparotomy, and laparoscopy was considered indicative of major AL. Drainage of an ischiorectal abscess and specified or unspecified drainage through the perineal region was not indicative of major AL, but was considered indicative of minor AL. If a patient met the criteria for both major and minor AL, they were considered to have major AL.

Patients with a stoma formation during the index surgery and those with subsequent intervention for AL on the same day as the index surgery (day 0) were excluded from the study because the minimum time interval recorded in the HES is 1 day, meaning that repeat operation on the same day cannot be identified separately from the index surgery. Patients with records indicating postoperative bleeding between days 0 and 30 after surgery were also excluded to avoid misclassifying bleeding as AL. In addition, patients with primary stoma formation were excluded because, given the limitations of the data set (no radiology reports or laboratory results), the authors felt it would not be possible to positively identify AL in these patients, and thus there was a risk of misclassification bias among this group. Although patients with primary stoma were excluded to avoid misclassification bias, codes related to refashioning of stoma were included to be increase the likelihood of identifying AL. Procedure coding is performed by clinical coders based on medical notes/operations notes and standards of coding are provided by NHS England. This exclusion criterion was included after discussions with surgeons experienced in managing AL. The authors acknowledge that this could lead to underreporting of early AL. However, given that within the HES data set it is not possible to distinguish between multiple procedures occurring in the same operation (for example hemicolectomy and stoma formation) or occurring in different operations on the same day, there was a risk that patients undergoing multiple procedures would be misclassified and the incidence of AL could be overestimated; thus, it was decided to err on the side of a conservative estimate.

Matched patients were drawn from patients undergoing left-sided colorectal surgery without evidence of AL described above from days 1 to 30 following surgery.

### Matching criteria

For all analyses, 1:1 exact matching was performed between patients developing AL and those without AL using the following factors:

- age at index surgery (by 10-year age band, including 18–29 years and ≥ 80 years)
- sex (male or female)
- date of colorectal surgery (± 30 days of the date of the index surgery)
- type of colorectal surgery procedure (left hemicolectomy, sigmoid or transverse colectomy, rectal resection)
- elective/non-elective status for colorectal surgery admission
- malignant or non-malignant indication for surgery.

Additional variables that were not matched were included as covariates in regression analyses.

The aim of matching in this way was to assess the difference in costs attributable to AL in excess of the cost of primary surgery and recovery, so a robust comparator group undergoing similar procedures without AL was needed to reduce potential bias from patients with other characteristics (that is, older, diabetic, or having more cancer).

### Outcomes of interest

The primary objective of this study was to compare the all-cause inpatient cost for surgical hospitalization and the number of subsequent admissions within 90 days of the index surgery between patients with and without AL after left-sided colorectal surgery.

Secondary objectives were to estimate the annual incidence rate of AL requiring intervention after left-sided colorectal surgery, within 30 days of surgery between 2018 and 2021; to describe the baseline characteristics, hospitalizations, and cumulative LOS (including both surgical and subsequent admissions) for patients with and without AL after left-sided colorectal surgery; and to further describe baseline characteristics, hospitalizations, cumulative LOS, and all-cause inpatient costs for patients with major and minor AL, each matched with patients without AL.

The study also had exploratory objectives, namely to compare healthcare resource use in patients with *versus* without AL; to investigate the association of overall AL, major AL, minor AL, and no AL on in-hospital mortality; and differences in discharge destination and postoperative infection rates for those with and without AL. Discharge destination was defined as discharge to place of residence or a nursing home. Postoperative infection was measured as a composite outcome, incorporating postoperative wound infection, pneumonia, and urinary tract infection.

The cost of AL has been calculated as a total all-cause cost, which includes the reimbursement provided to the hospital for the index surgery and subsequent readmission, as well as additional costs to the hospital of intensive care unit (ICU) stays. England has a single payer system; costs are either reimbursed by local commissioning groups using the agreed national tariff, reimbursed by central government for highly specialized care (for example, cardiothoracic surgery), or, as in the case with ICUs, negotiated locally with local commissioning groups. Where costs are locally reimbursed there is no national tariff; therefore, the ICU costs from the National Schedule of Costs are used as a proxy. All payers (local commissioners and central government commissioners) are funded through taxation; therefore, this study takes the national payer or healthcare system perspective.

### Ethics approval

As stated in the data-sharing agreement between NHS Digital and CorEvitas Specialty EMR Data division (legally traded as Health iQ), the data used in this study are in an anonymized structured format, containing no patient identifiable information; therefore, ethics approval was not required because no confidential data were being shared.

### Statistical analysis

This study was powered for the primary outcome of total cost, comparing patients with and without AL in the 90 days following index surgery based on an expected population of 1000 matched patients and a difference in cost of €20,400^23^. The details of the sample size calculation are reported in the *supplementary methods*, along with the Great British Pound (GBP) amount used. Statistical tests and *P*-values for all other outcomes and comparisons (for example, major AL *versus* without AL; minor AL *versus* without AL) are considered exploratory, and no multiple comparison adjustment is considered.

Baseline characteristics and outcomes are described as the absolute number and percentage of patients for categorical variables and as the mean, median, or range (minimum–maximum) for continuous variables.

Costs for inpatient care were generated from the NHS Digital Healthcare Resource Group (HRG) payment grouper, which maps each patient’s diagnosis (International Classification of Diseases, Tenth Revision) and procedure (OPCS Classification of Interventions and Procedures version 4) codes to an HRG code. The tariff payments used in this analysis are for the period 2022–2023. Costs for ICU admissions were calculated using the National Cost Collection Schedule of NHS costs, based on the 2020–2021 financial year, which were inflated to the 2022 financial year using the NHS cost inflation index. Costs are presented in euro based on purchasing power parity for 2022^23^.

All analyses comparing costs, hospitalization rates, and clinical outcomes reported in this paper were based on matched patients with and without AL. For all analyses, the adjusted difference (Charlson co-morbidity index (CCI) score and geographical region) for each outcome are provided; absolute descriptive values for each group are also provided.

Generalized linear models (GLM) with gamma family and identity link were used to compare costs for matched patients with and without AL. GLM with negative binomial regression family and identity link were used to estimate the excess cumulative LOS associated with AL. However, due to the sparseness of the data for cumulative LOS, the GLM did not converge; therefore, 0.5 was set to all factors in this model as a starting value for the estimates of the model coefficients. As has been done in previous research, the choice for an informed starting value (instead of the default, often all zeros) provides the algorithm with a closer point in parameter space to begin with, thus reducing the chance of divergence and numerical instability in fitted values. Conditional logistic regression was used to investigate the association between AL and clinical outcomes (inpatient mortality, discharge destination, and postoperative infection), accounting for the matched setting.

All comparative analyses were conducted on matched patients with *versus* without AL and repeated to consider any difference between patients with each of minor and major AL *versus* those with without AL. All multivariate models were adjusted for the CCI and geographical region.

## Results

### Study population

**Fig. 1:**
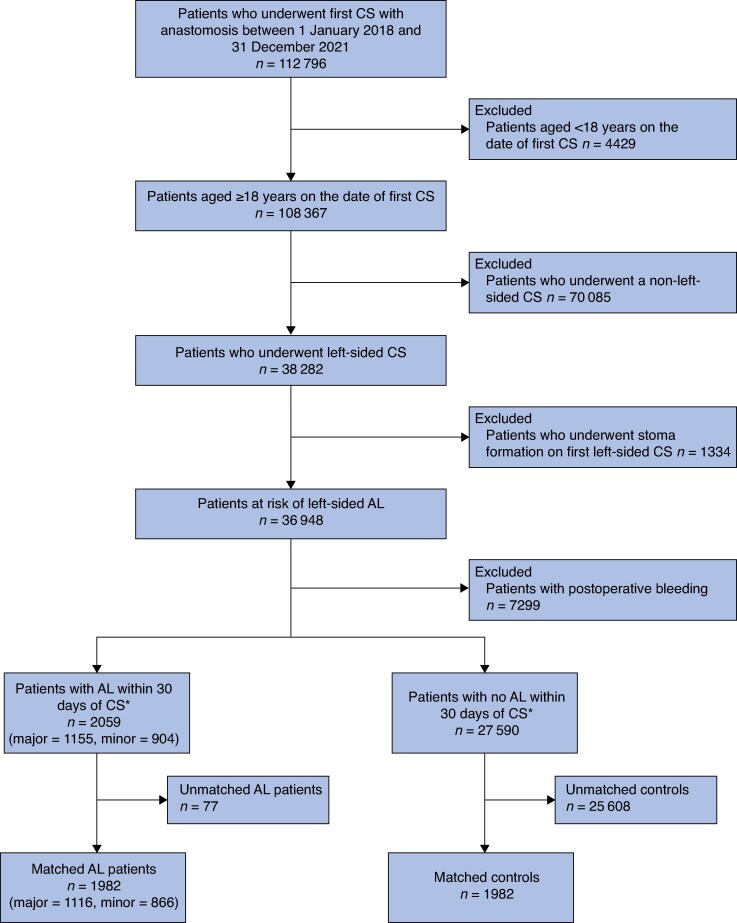
Patient population study flow chart *Within 30 days was defined as from day 1 after CS to day 30. Excludes AL coded on the day of surgery (day 0). CS, colorectal surgery; AL, clinical anastomotic leak.

Overall, 112 796 patients underwent any colorectal surgery between 1 January 2018 and 31 December 2021, 38 282 of which were left hemicolectomies, sigmoid, or rectal operations (*Fig. 1*). Among these patients, 36 948 adult patients who underwent left-sided colorectal surgery with anastomosis without stoma formation on their index surgery were identified. After excluding those patients with codes for bleeding complications, done to avoid overestimation of AL using reoperation codes, 2059 patients with AL and 27 590 patients without AL were identified, resulting in 1982 exact matched patients. Of the patients with AL, 1116 had major AL and 866 had minor AL.

Patients were not matched on specific co-morbidities; however, the mean CCI score was similar between patients with and without AL (2.2 and 1.9, respectively).

Baseline data for matched patients are summarized in *Table 1*.

**Table 1:** Baseline demographics and characteristics of matched patients with and without AL

	Overall cohort		Major AL		Minor AL	
	AL (+) (*n* = 1982)	AL (–) (*n* = 1982)	AL (+) (*n* = 1116)	AL (–) (*n* = 1116)	AL (+) (*n* = 866)	AL (–) (*n* = 866)
Age group*						
18–29 years	70 (3.5%)	70 (3.5%)	28 (2.5%)	28 (2.5%)	42 (4.8%)	42 (4.8%)
30–39 years	107 (5.4%)	107 (5.4%)	44 (3.9%)	44 (3.9%)	63 (7.3%)	63 (7.3%)
40–49 years	171 (8.6%)	171 (8.6%)	104 (9.3%)	104 (9.3%)	67 (7.7%)	67 (7.7%)
50–59 years	375 (18.9%)	375 (18.9%)	218 (19.5%)	218 (19.5%)	157 (18.1%)	157 (18.1%)
60–69 years	549 (27.7%)	549 (27.7%)	323 (28.9%)	323 (28.9%)	226 (26.1%)	226 (26.1%)
70–79 years	533 (26.9%)	533 (26.9%)	303 (27.2%)	303 (27.2%)	230 (26.6%)	230 (26.6%)
≥ 80 years	177 (8.9%)	177 (8.9%)	96 (8.6%)	96 (8.6%)	81 (9.4%)	81 (9.4%)
Sex*						
Male	1188 (59.9%)	1188 (59.9%)	665 (59.6%)	665 (59.6%)	523 (60.4%)	523 (60.4%)
Female	794 (40.1%)	794 (40.1%)	451 (40.4%)	451 (40.4%)	343 (39.6%)	343 (39.6%)
**Ethnicity**						
White	1572 (79.3%)	1583 (79.9%)	905 (81.1%)	913 (81.8%)	667 (77.0%)	670 (77.4%)
Black	35 (1.8%)	29 (1.5%)	20 (1.8%)	14 (1.3%)	15 (1.7%)	15 (1.7%)
South Asian	33 (1.7%)	28 (1.4%)	20 (1.8%)	15 (1.3%)	13 (1.5%)	13 (1.5%)
Mixed	14 (0.7%)	9 (0.5%)	6 (0.5%)	2 (0.2%)	8 (0.9%)	7 (0.8%)
Other	52 (2.6%)	40 (2.0%)	26 (2.3%)	21 (1.9%)	26 (3.0%)	19 (2.2%)
Unknown	276 (13.9%)	293 (14.8%)	139 (12.5%)	151 (13.5%)	137 (15.8%)	142 (16.4%)
Left-sided colorectal surgery type*						
Colectomy	581 (29.3%)	581 (29.3%)	371 (33.2%)	371 (33.2%)	210 (24.2%)	210 (24.2%)
Hemicolectomy	555 (28.0%)	555 (28.0%)	222 (19.9%)	222 (19.9%)	333 (38.5%)	333 (38.5%)
Rectal resection	845 (42.6%)	845 (42.6%)	522 (46.8%)	522 (46.8%)	323 (37.3%)	323 (37.3%)
Other	1 (0.1%)	1 (0.1%)	1 (0.1%)	1 (0.1%)	0 (0%)	0 (0%)
**Indication for surgery**						
Malignant	863 (43.5%)	829 (41.8%)	523 (46.9%)	483 (43.3%)	340 (39.3%)	346 (40.0%)
Non-malignant	1119 (56.5%)	1153 (58.2%)	593 (53.1%)	633 (56.7%)	526 (60.7%)	520 (60.0%)
Index admission type*						
Elective	671 (33.9%)	671 (33.9%)	334 (29.9%)	334 (29.9%)	337 (38.9%)	337 (38.9%)
Non-elective	1311 (66.1%)	1311 (66.1%)	782 (70.1%)	782 (70.1%)	529 (61.1%)	529 (61.1%)
**Charlson co-morbidity index score**						
Mean(s.d.)	2.2(2.1)	1.9(2)	2.2(2)	1.9(2)	2.3(2.2)	1.8(2)
Median (i.q.r.)	2 (0–3)	2 (0–3)	2 (0–3)	2 (0–3)	2 (0–3)	2 (0–3)
Range	12 (0–12)	13 (0–13)	10 (0–10)	12 (0–12)	12 (0–12)	13 (0–13)
**No. of co-morbidities**						
0	587 (29.6%)	698 (35.2%)	319 (28.6%)	396 (35.5%)	268 (30.9%)	302 (34.9%)
≥ 1	1395 (70.4%)	1284 (64.8%)	797 (71.4%)	720 (64.5%)	598 (69.1%)	564 (65.1%)

Values are *n* (%) unless otherwise stated. *Matching criteria. AL, anastomotic leak; AL (+), with anastomotic leak; AL (–), without anastomotic leak; s.d., standard deviation; i.q.r., interquartile range.

The most common (42.6%) index surgery of patients developing AL was rectal resection (*Table 1*). Of those patients undergoing colectomy as their initial surgery and subsequently developing AL, more developed major than minor AL (33.2 *versus* 24.2%, respectively). Conversely, after hemicolectomy, a lower proportion of patients developed major than minor AL (19.9 *versus* 38.5%, respectively).

A lower proportion of patients developing major AL underwent elective procedures compared with those developing minor AL (29.9 *versus* 38.9%, respectively), indicating major leaks were overrepresented in the acute surgery setting.

### All-cause inpatient costs within the 90-day follow-up

Patients with AL had a significantly higher estimated mean all-cause inpatient cost than patients without AL (adjusted difference €11,728^23^; 95% confidence interval (c.i.) €11 562 to €14 808; *P* < 0.001; *Table 2*).

**Table 2:** All-cause inpatient costs within 90 days of follow-up and surgery hospitalization costs (between 1 January 2018 and 31 December 2021) for matched patients with and without AL

Resource use metric	HCRU absolute values*						HCRU statistical modelling: AL (+) *versus* AL (–)*,†		
	Overall patients with AL		Major AL		Minor AL		Overall patients with AL	Major AL	Minor AL
	AL (+) (*n* = 1982)	AL (–) (*n* = 1982)	AL (+) (*n* = 1116)	AL (+) (*n* = 1982) *versus* AL (–) (*n* = 1982)	AL (+) (*n* = 866)	AL (–) (*n* = 866)			
**Total all-cause cost including index surgery hospitalization, ICU, and subsequent hospital admission within 90 days of follow-up (€)**									
Mean(s.d.)	24 356 (32 837)	12 311 (13 304)	27 003 (39 920)	11 718(12 489)	20 975 (20 020)	13 077 (14 257)	11 728 (10 313–13 209)‡	14 478 (12 602–16 506)‡	8066 (6403–9874)‡
Median (i.q.r.)	16 739 (10 750–26 983)	8403 (6740–12 961)	18 361 (11 594–28 932)	8214 (6709–12 549)	14 675 (9883–23 996)	8600 (6852–13 330)			
**Index surgery hospitalization costs, including ICU admissions (€)**									
Mean(s.d.)	20 198 (31 528)	11 239 (12 083)	22 099 (38 599)	10 676 (10 971)	17 753 (18 624)	11 969 (13 356)			
Median (i.q.r.)	12 081 (7545–22 229)	7770 (6617–11 468)	12 415 (7262–24 049)	7653 (6544–11 138)	11 790 (8015–20 497)	8012 (6712–12 069)			
**Subsequent admission costs, including ICU admissions (€)**									
Mean(s.d.)	4034(10 115)	1049(4622)	4857(11 938)	1059(5404)	2979(6995)	1035(3363)			
Median (i.q.r.)	647 (0–4205)	0 (0–500)	802 (0–5763)	0 (0–480)	419 (0–3115)	0 (0–522)			

*Absolute values are calculated based on matched patients AL *versus* matched controls for overall AL patients, and matched major/minor AL patients *versus* matched controls for major/minor AL; modelling data are calculated on the matched overall/major/minor patients with AL *versus* the matched AL control patients only. †Data show the adjusted difference; values in parentheses are 95% confidence intervals. All models were adjusted for the CCI and geographical region. For all-cause inpatient cost, the adjustment factors were as follows. Region: East of England, reference; London, 2227; Midlands, 241; North East and Yorkshire, −1563; North West, −953, South East, 619; South West, −1746; outside of England, −1234 (*P* > 0.05 for all); unknown, 11,502 (*P* = 0.023). CCI: score 0, reference; score 1, 1,548 (*P* = 0.2); score 2, −2,043 (*P* = 0.002); score 3, −234 (*P* = 0.8); score 4, 2,099 (*P* = 0.2); score 5, 852 (*P* = 0.5); score ≥6, 6,716 (*P* < 0.001). For all-cause number of hospitalizations, the adjustment factors were as follows. Region: East of England, reference; London, 0.37 (*P* = 0.002); Midlands, 0.18; North East and Yorkshire, 0.08; North West, −0.04, South East, 0.07; South West, 0.12; outside of England −0.45; Unknown, 0.12 (*P* > 0.05 for all). CCI: score 0, reference; score 1, −0.05 (*P* = 0.6); score 2, 0.47; score 3, 0.51; score 4, 0.35; score 5, 0.59; score ≥6, 0.98 (*P* < 0.05 for all). AL, anastomotic leak; HCRU, healthcare resource utilization; AL (+), with anastomotic leak; AL (–), without anastomotic leak; ICU, intensive care unit; s.d., standard deviation; i.q.r., interquartile range; CCI, Charlson co-morbidity index. ‡*P* < 0.05.

Compared with patients without AL, mean all-cause inpatient costs were significantly higher for patients with major AL (adjusted difference €10 313; 95% c.i. €12 602 to €16 506; *P* < 0.001), and for those with minor AL (adjusted difference €8066; 95% c.i. €6403 to €9874; *P* < 0.001; *Table 2*).

Patients with all grades of AL had numerically higher (statistical tests not performed) mean index surgery hospitalization costs (including ICU costs) than those without AL: €20 198 *versus* €11 239 for overall (major and minor) AL *versus* without AL, respectively; €22 099 *versus* €10 676 for major AL *versus* matched patients without AL, respectively; and €17 753 *versus* €11 969 for minor AL *versus* matched patients without AL, respectively. Mean costs for subsequent hospitalizations were also numerically higher for overall AL than without AL (€4034 *versus* €1049, respectively); for major AL than without AL (€4857 *versus* €1059, respectively); and for minor AL than without AL (€2979 *versus* €1035, respectively; *Table 2*).

All-cause inpatient costs in GBP are provided in *Table S3*.

### Estimated annual AL incidence rate

It is well established that the incidence of AL varies depending on the surgical site and the definition of AL^10^. In addition, as noted above, incidence rates reported in the literature are highly variable.^7^ The aim of this study was to determine the incidence of AL using proxy procedure codes from the NHS HES database.

Of all patients at risk of left-sided AL (that is, those undergoing left-sided colorectal surgery with anastomosis, without stoma formation on the index colorectal surgery) during the study period, 5.57% developed an AL requiring intervention. A reduction in the overall number of colorectal surgeries was seen in the 2020 calendar year. During 2020–2021 there was a notable increase in the proportion of colorectal surgery patients developing AL (*Table 3*).

**Table 3:** Estimated incidence rates of AL in England (2018–2021)

	No. of left-sided colorectal surgeries with anastomosis	No. of patients with AL (%)	Estimated incidence of AL per 1000 surgeries
Overall	36 948	2059 (5.57%)	55.7
2018	9658	467 (4.84%)	48.3
2019	9660	481 (4.98%)	49.7
2020	7764	487 (6.27%)	62.7
2021	9866	626 (6.35%)	63.4

AL, anastomotic leak.

### All-cause hospitalizations and cumulative LOS

Data on all-cause hospitalizations and cumulative LOS for matched patients are summarized in *Table 4*.

**Table 4:** Summary of hospitalizations, cumulative length of hospital stay, and clinical outcomes for matched patients with and without AL

Resource use metric	HCRU absolute values*						HCRU statistical modelling: AL (+) *versus* AL (–)*†		
	Overall AL patients		Major AL		Minor AL		Overall patients with AL	Major AL	Minor AL
	AL (+) (*n* = 1982)	AL (–) (*n* = 1982)	AL (+) (*n* = 1116)	AL (+) (*n* = 1982) *versus* AL (–) (*n* = 1982)	AL (+) (*n* = 866)	AL (–) (*n* = 866)			
**No. of all-cause hospitalizations‡**									
Mean(s.d.)	2.3(2.2)	1.9(1.7)	2.2(1.8)	1.9(1.9)	2.3(2.7)	1.8(1.5)	Adjusted difference		
							0.37 (0.26–0.49)**	0.30 (0.17–0.44)**	0.46 (0.31–0.62)**
Median (i.q.r.)	2.0 (1.0–3.0)	1.0 (1.0–2.0)	2.0 (1.0–3.0)	1.0 (1.0–2.0)	2.0 (1.0–3.0)	1.0 (1.0–2.0)			
**No. of subsequent hospitalizations during follow-up**									
No. of patients with subsequent admission(s)	1167 (58.9%)	778 (39.3%)	675 (60.5%)	447 (40.1%)	492 (56.8%)	331 (38.2%)			
Mean(s.d.) no. of subsequent admissions	1.3(2.2)	0.9(1.7)	1.2(1.8)	0.9(1.9)	1.3(2.7)	0.8(1.5)			
Median (i.q.r.) no. of subsequent admissions	1.0 (0.0–2.0)	0.0 (0.0–1.0)	1.0 (0.0–2.0)	0.0 (0.0–1.0)	1.0 (0.0–2.0)	0.0 (0.0–1.0)			
**Cumulative LOS for all-cause hospitalizations (days)**									
Mean(s.d.)	30.1(30.5)	13.7(18.2)	29.7(32.3)	12.9(17.8)	30.5(28.1)	14.7(18.7)	Adjusted difference		
							15.93 (14.24–17.61)**	15.66 (13.53–17.78)**	16.28 (13.79–18.76)**
Median (i.q.r.)	21.0 (13.0–35.0)	7.0 (5.0–15.0)	20.0 (12.0–35.0)	7.0 (4.0–13.0)	22.0 (14.0–36.0)	8.0 (5.0–16.0)			
**LOS for surgery hospitalization (days)**									
Mean(s.d.)	23.3(27.6)	11.9(15.3)	22.1(29.2)	11.3(15.7)	24.8(25.4)	12.7(14.8)			
Median (i.q.r.)	15.0 (7.0–28.0)	7.0 (4.0–13.0)	13.0 (6.0–28.0)	7.0 (4.0–12.0)	17.0 (10.0–29.0)	7.0 (5.0–15.0)			
**LOS for subsequent hospitalizations during follow-up**§ **(days)**									
Mean(s.d.)	6.8(15.8)	1.9(7.6)	7.6(16.9)	1.7(6.9)	5.6(14.0)	2.0(8.4)			
Median (i.q.r.)	0.0 (0.0–7.0)	0.0 (0.0–0.0)	0.0 (0.0–10.0)	0.0 (0.0–0.0)	0.0 (0.0–5.0)	0.0 (0.0–0.0)			
**In-hospital mortality**									
During surgery hospitalization or within 90 days	158 (8.0%)	87 (4.4%)	102 (9.1%)	40 (3.6%)	56 (6.5%)	47 (5.4%)	Adjusted OR		
							0.58 (0.4–0.85)**	0.41 (0.24–0.7)**	0.86 (0.5–1.47)
**Discharge**									
No. of patients alive at discharge	1827	1894	1014	1075	813	819			
No. of patients discharged to place of residence¶	1725 (94.4%)	1838 (97.0%)	957 (94.4%)	1048 (97.5%)	768 (94.5%)	790 (96.5%)	Adjusted OR		
							0.58 (0.4–0.85)**	0.41 (0.24–0.7)**	0.86 (0.5–1.47)
**Postoperative infection**#									
Any postoperative infection	1176 (59.3%)	552 (27.9%)	721 (64.6%)	552 (49.5%)	455 (52.5%)	552 (63.7%)	Adjusted OR		
							3.66 (3.14–4.27)**	4.7 (3.8–5.81)**	2.68 (2.15–3.34)**
Surgical wound infection	769 (38.8%)	264 (13.3%)	524 (47.0%)	148 (13.3%)	245 (28.3%)	116 (13.4%)			
Pneumonia	512 (25.8%)	229 (11.6%)	273 (24.5%)	124 (11.1%)	239 (27.6%)	105 (12.1%)			
Urinary tract infection	281 (14.2%)	210 (10.6%)	160 (14.3%)	117 (10.5%)	121 (14.0%)	93 (10.7%)			

Values are *n* (%) unless otherwise stated. *Absolute values were calculated based on matched patients AL *versus* matched controls for overall patients with AL, and matched major/minor AL patients *versus* matched controls for major/minor AL; modelling data are calculated on the matched overall/major/minor AL patients *versus* matched AL control patients only. †Data show the adjusted difference or adjusted OR; values in parentheses are 95% confidence intervals. All models were adjusted for the Charlson co-morbidity index and geographical region. ‡Includes surgery hospitalization and subsequent admissions, including intensive care unit admissions. §Each patient will have a mean LOS across multiple admissions. The LOS across all subsequent hospital admissions has been summed and reported as patient-level metrics of total LOS. ¶This follows a complete case analysis; all entries marked as unknown were removed. Models on discharge were not adjusted by geographical region because there was insufficient convergence to model this variable. #Infection categories were not mutually exclusive; patients were considered for the binary classification if they had any of the analysed infections, regardless of the number of times that was recorded. AL, anastomotic leak; HCRU, healthcare resource utilization; AL (+), with anastomotic leak; AL (–), without anastomotic leak; s.d., standard deviation; i.q.r., interquartile range; LOS, length of hospital stay; OR, odds ratio. **P < 0.05.

Patients with any grade of AL had significantly more mean all-cause hospitalizations (that is, the mean number of hospitalizations for any reason, among all patients) than patients without AL (adjusted difference 0.37; 95% c.i. 0.26 to 0.49; *P* < 0.001). Mean all-cause hospitalizations were significantly higher for patients with major AL *versus* patients without AL (adjusted difference 0.30; 95% c.i. 0.17 to 0.44; *P* < 0.001), and for patients with minor AL *versus* patients without AL (adjusted difference 0.46; 95% c.i. 0.31 to 0.62; *P* < 0.001).

Patients with AL also had a significantly higher mean cumulative LOS than patients without AL (adjusted difference 15.93 days; 95% c.i. 14.24 to 17.61 days; *P* < 0.001). In addition, mean cumulative LOS was significantly higher for patients with major (adjusted difference 15.66 days; 95% c.i. 13.53 to 17.78 days; *P* < 0.001) and minor AL (adjusted difference 16.28 days; 95% c.i. 13.79 to 18.76 days; *P* < 0.001) than for patients without AL.

### Clinical outcomes

Clinical outcomes data for matched patients are summarized in *Table 4*. Patients with AL had significantly higher odds of dying in hospital within 90 days of surgery than patients without AL (adjusted odds ratio (aOR) 2.17; 95% c.i. 1.56 to 3.02; *P* < 0.001). This was particularly the case for those developing major AL, of whom a larger proportion died in hospital compared with patients without AL (aOR 3.29; 95% c.i. 2.05 to 5.27; *P* < 0.001). There was no significant difference in the odds of patients with minor AL dying compared with patients without AL (aOR 1.3; 95% c.i. 0.8 to 2.12; *P* = 0.292).

Patients with AL were less likely to be discharged to their place of residence than patients without AL (aOR 0.58; 95% c.i. 0.4 to 0.85; *P* = 0.004). This was particularly the case for patients with major AL *versus* those without AL (aOR 0.41; 95% c.i. 0.24 to 0.7; *P* = 0.001). There was no significant difference in the odds of being discharged to their place of residence between patients with minor AL and those without AL (aOR 0.86; 95% c.i. 0.5 to 1.47; *P* = 0.573).

Patients with AL were more likely to develop a postoperative infection than patients without AL (aOR 3.66; 95% c.i. 3.14 to 4.27; *P* < 0.001). This was also true for patients with major AL (aOR 4.7; 95% c.i. 3.8 to 5.81; *P* < 0.001) and minor AL (aOR 2.68; 95% c.i. 2.15 to 3.34; *P* < 0.001).

## Discussion

This study demonstrated that the development of an AL is associated with poorer clinical and economic outcomes, even in patients with minor leaks, in whom LOS was also increased despite the fact that in-hospital mortality was not significant. Mean all-cause inpatient costs were significantly higher among patients with than without AL.

Overall, mean all-cause hospitalizations and cumulative LOS were significantly higher in patients with than without AL. Patients with AL also had a significantly higher in-hospital mortality rate within the first 90 days after surgery, lower odds of being discharged home, and a higher risk of developing infection. Furthermore, any additional LOS because of AL may both negatively impact patient outcomes and reduce potential revenue for the hospital because while the bed is occupied the hospital is unable to treat another patient.

The findings of this study agree with analyses in similar European settings, showing that the development of AL is associated with poorer economic outcomes, with increased LOS and the need for additional surgery and intensive care treatment being among the greatest drivers of cost increases^16–18,20^. However, estimates of incremental cost increases due to AL are highly variable. For example, even in a patient group undergoing lower resection for colorectal cancer, costs across countries ranged from €7289 to €83,633, reflective of differences in national healthcare costs and methodologies for estimating costs^21^. In a previous study in 2013^18^, the actual cost of AL was estimated to be £17 220 (€18,942^23^), but that study focused only on anterior resection whereas the present study included all left-sided colorectal surgery, including left hemicolectomy, sigmoid or transverse colectomy, rectal resection. This difference in surgical procedures could explain the cost differences.

The overall AL incidence reported in this study was lower than in a previous UK study^18^ of 285 patients (5.57 *versus* 10.9%, respectively) and the 2017 ESCP Left Colon, Sigmoid and Rectal Resections Audit^8^ (Northern Europe, including the UK; 5.57 *versus* 9.2%, respectively). This difference may be due to how AL is identified and data were collected; in the ESCP audit^8^, AL was defined as radiologically or clinically proven ALs and/or intra-abdominal or pelvic collection. However, in the present retrospective study, AL was identified through administrative clinical coding only. Because there was no specific diagnosis or procedural code for AL, it may be underreported in the present analysis. This study encompasses a wider range of underlying pathologies as indicative of AL, including minor AL not requiring further surgical intervention, in the absence of a diagnostic code to identify patients, whereas the previous UK study^18^ used laparotomy within 28 days of index surgery as a surrogate for AL. The location of the anastomosis may also be a consideration, with rectal anastomoses having a higher risk of AL than colonic anastomoses; if studies include different case mixes, they may have correspondingly different mean leak rates^5^.

Interestingly, the present data highlighted an increase in the estimated AL rate requiring intervention during 2020–2021. This was despite a reduction in the number of procedures in 2020, likely due to the impact of COVID-19. The increase in the rate during the last 2 years of the study period may be due to a higher number of urgent procedures and surgeries on later-stage disease after the pandemic; however, this generally represents a minority of the total cases^26,27^. A further possibility is that, rather than a significant change in the AL rate itself, the threshold for intervention reduced during that time. Determining the specific contributory factors to the increased rate requires further study, and more recent data would be useful to see whether this trend has continued. Regardless, the poorer clinical and economic burden associated with even minor AL demonstrated in the present study underlines the importance of reducing the occurrence of this complication after left-sided colorectal surgery.

This study highlights both the importance of incorporating cost data into future trials to further explore the economic burden of AL and optimizing surgical techniques, possibly via the use of novel stapling procedures and enhanced recovery after surgery (ERAS) pathways to reduce the risk of this form of complication.

This study is robust in its use of a national database that covers all NHS-funded care in England, providing a substantial cohort of patients developing AL, most (96%, 1982) of whom could be exactly matched to similar patients without AL, with additional adjustment for CCI and region. Exact matching was chosen over propensity score matching because the data set used in this study contained only limited covariates that could be controlled for.

This study has several limitations that need to be addressed in future studies. The present retrospective study relied on HES data, which only capture events within, and data input during secondary care. Therefore, mortality data outside of the hospitalized period(s) would not be captured.

AL is not directly recorded in HES data; therefore, proxies for AL were used, which could result in inaccuracies in AL incidence (for example, if reoperation due to incomplete margins for resection of malignancy occurred within 30 days of colorectal surgery, this could be misclassified as major AL). There is also the potential for misclassification (for example, relaparotomy for bleeding or non-leak indications), especially given the lack of radiology reporting and laboratory results, and the authors relied on the recording of procedures that align with ISREC grades B and C AL. Although there is no generally accepted definition of AL, a consensus on its definition was reported in 2020^5^, and standardization based on this would ensure consistency and comparability in future work.

The coding policies of NHS providers, as well as changing health information systems may also introduce variation in coding across providers. In addition, misclassification of patients cannot be controlled for in retrospective data; misclassification is likely to include those with suspected AL who may have a diagnostic laparoscopy or an open-and-close relaparotomy and do not have a definitive AL. According to the definition used in the present study, these patients will be classed as having AL. Requiring colorectal surgery and AL to be separated in time is important to prevent complex surgeries resulting in multiple procedure codes from being misclassified as major AL. However, specifying this may mean some patients who experience AL on the same day as the initial operation are misclassified as controls. Because patients were not matched on specific co-morbidities, certain conditions were overrepresented in the group of patients with AL. This could potentially have resulted in a slight overestimation of costs.

This study spans the period when the UK health service was impacted by COVID-19 management, which likely affected the number of colorectal surgery procedures performed in 2020, as discussed previously. This study was also designed to capture short-term costs and complications; therefore, it does not capture longer-term morbidity. A prospective study is required to evaluate longer-term economic and societal costs. Although not explored in the present study, it may also be of interest to understand differences in time to diagnosis for major and minor AL and the proportions of patients diagnosed during initial admission.

This study demonstrated that the development of an AL is associated with poorer clinical outcomes, increased HCRU, and a substantial economic burden, even in patients with minor leaks. The cost of treatment increases with the severity of AL, emphasizing the need to mitigate the risk of its occurrence after left-sided colorectal surgery.

## Supplementary Material

zrag049_Supplementary_Data

## Data Availability

Relevant data are available from the corresponding author upon reasonable request.
