# Supplementary material for: Costs associated with anastomotic leak after left-sided colorectal surgery: retrospective real-world study in England
Source: BJS Open. 2026 May 20;10(3):zrag049. doi: 10.1093/bjsopen/zrag049 (PMC13188152; doi:10.1093/bjsopen/zrag049)
Supplement: zrag049_Supplementary_Data [file zrag049_supplementary_data.docx]

**The costs associated with anastomotic leak after left-sided colorectal surgery: a retrospective real-world study in England**

Authors: Julia Glover-Kirtland,^1^ Cindy Tong,^2^ Melek Pinar Bosut,^3^ Niels-Derrek Schmitz,^3^ Sara Joao Carvalho,^4^ Caoimhe T Rice,^4^ Dion G Morton,^5^ Elizabeth Li,^5^ Thomas D Pinkney^5^

Corresponding author: Julia Glover-Kirtland

Affiliations:

1Johnson & Johnson MedTech, Berkshire, UK.

2Johnson & Johnson MedTech, New Brunswick, NJ, USA.

3Johnson & Johnson Medical GmbH, Norderstedt, Germany.

4 Thermo Fisher Scientific, London, UK.

5University of Birmingham, Academic Department of Surgery, Birmingham, UK

**Supplementary Materials - Index**

| **Supplementary Methods** |  |
| --- | --- |
| Sample size calculation | *page 2* |
| **Supplementary Figures and Tables** |  |
| Supplementary Figure 1: Study design | *page 3* |
| Supplementary Table 1: OPCS-4 and ICD-10 codes for left-sided colorectal surgery | *page 4* |
| Supplementary Table 2: OPCS-4 codes for markers of anastomotic leakage | *page 6* |
| Supplementary Table 3: All-cause inpatient costs within 90 days of follow-up and surgery hospitalisation costs (between 1 January 2018–31 December 2021) for matched patients with AL and without AL in GBP | *Page 7* |
| **References** | *page 8* |

**Supplementary Methods**

Assuming an AL rate of 1% (obtained from published estimates), a population of >80,000 patients undergoing left-sided colorectal surgery would lead to >800 patients with AL. Assuming, 1,000 patients in each matched group (with AL and without AL), with a one-sided t-test and α=0.05 and assuming the difference in costs (including index hospitalisation and re-admission) for those with and without AL to be 65% (the lowest cost difference in the literature). Those with AL would have an average cost of £20,000 (€20,400) and standard deviation of £10,000 (€10,200), which gives a statistical power of >99% for the primary outcome of total cost.

**Supplementary Figures and Tables**

**Supplementary Figure 1: Study design**


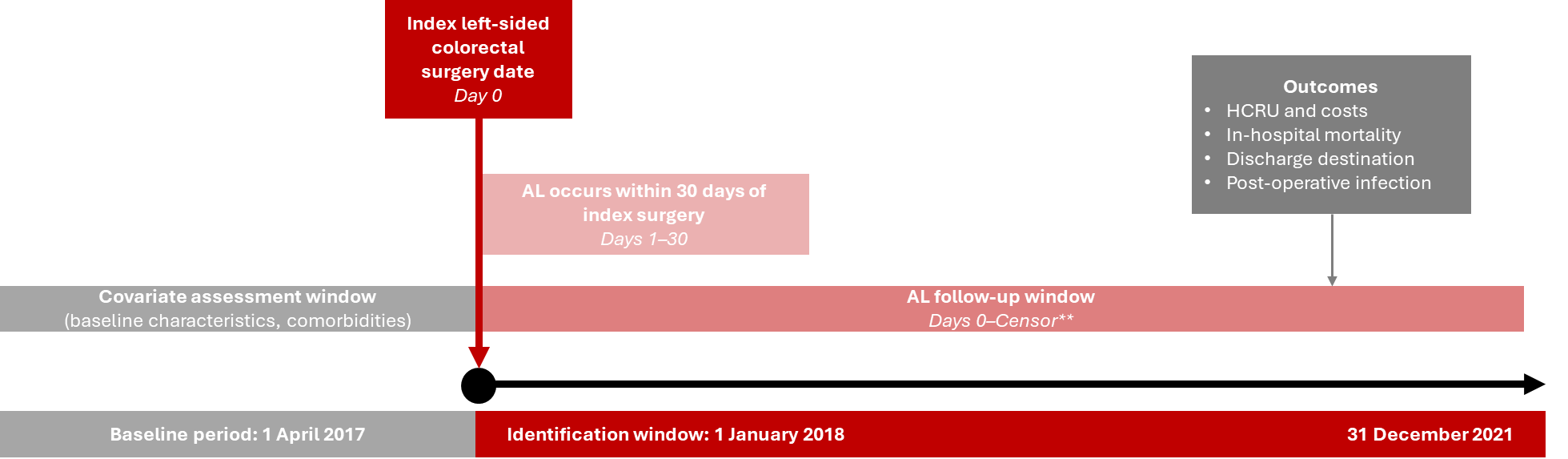


*AL, anastomotic leak; HCRU, healthcare resource utilisation*

**Defined using Office of Population Census and Surveys Classification of Interventions and Procedures version 4
** Censor defined as 120 days after index date, end of admission for those hospitalised at 120 days, date of inpatient death, or 31 December 2021, whichever occurred first*

**Supplementary** **Table 1: OPCS-4 and ICD-10 codes for left-sided colorectal surgery**

| Procedure codes (OPCS-4) | |
| --- | --- |
| Code | Description |
| H081 | Transverse colectomy and end to end anastomosis |
| H082 | Transverse colectomy and anastomosis of ileum to colon |
| H083 | Transverse colectomy and anastomosis NEC |
| H086 | Transverse colectomy and end to side anastomosis |
| H088 | Other specified excision of transverse colon |
| H089 | Unspecified excision of transverse colon |
| H091 | Left hemicolectomy and end to end anastomosis of colon to rectum |
| H092 | Left hemicolectomy and end to end anastomosis of colon to colon |
| H093 | Left hemicolectomy and anastomosis NEC |
| H096 | Left hemicolectomy and end to side anastomosis |
| H098 | Other specified excision of left hemicolon |
| H099 | Unspecified excision of left hemicolon |
| H101 | Sigmoid colectomy and end to end anastomosis of ileum to rectum |
| H102 | Sigmoid colectomy and anastomosis of colon to rectum |
| H103 | Sigmoid colectomy and anastomosis NEC |
| H106 | Sigmoid colectomy and end to side anastomosis |
| H108 | Other specified excision of sigmoid colon |
| H109 | Unspecified excision of sigmoid colon |
| H111 | Colectomy and end to end anastomosis of colon to colon NEC |
| H113 | Colectomy and anastomosis NEC |
| H116 | Colectomy and end to side anastomosis NEC |
| H118 | Other specified other excision of colon |
| H119 | Unspecified other excision of colon |
| H133 | Bypass of colon by anastomosis of transverse colon to sigmoid colon |
| H134 | Bypass of colon by anastomosis of transverse colon to rectum |
| H135 | Bypass of colon by anastomosis of colon to rectum NEC |
| H138 | Other specified bypass of colon |
| H139 | Unspecified bypass of colon |
| H291 | Subtotal excision of colon and rectum and creation of colonic pouch and anastomosis of colon to anus |
| H292 | Subtotal excision of colon and rectum and creation of colonic pouch NEC |
| H293 | Subtotal excision of colon and creation of colonic pouch and anastomosis of colon to rectum |
| H294 | Subtotal excision of colon and creation of colonic pouch NEC |
| H298 | Other specified subtotal excision of colon |
| H299 | Unspecified subtotal excision of colon |
| H333 | Anterior resection of rectum and anastomosis of colon to rectum using staples |
| H334 | Anterior resection of rectum and anastomosis NEC |
|  |  |
| **Diagnosis codes (ICD-10)** | |
| **Code** | **Malignant diagnoses requiring left-sided colon operation** |
| D01.2 | Carcinoma in situ: Rectum |
| D12.3 | Benign neoplasm: Transverse colon |
| D12.4 | Benign neoplasm: Descending colon |
| D12.5 | Benign neoplasm: Sigmoid colon |
| D12.6 | Benign neoplasm: Colon unspecified |
| D12.7 | Benign neoplasm: Rectosigmoid junction |
| D12.8 | Benign neoplasm: Rectum |
| D12.8 | Benign neoplasm: Rectum |
| C18.4 | Malignant neoplasm: Transverse colon |
| C18.5 | Malignant neoplasm: Splenic flexure |
| C18.6 | Malignant neoplasm: Descending colon |
| C18.7 | Malignant neoplasm: Sigmoid colon |
| C18.9 | Malignant neoplasm: Colon unspecified |
| C19 | Malignant neoplasm of rectosigmoid junction |
| C20 | Malignant neoplasm of rectum |
| C21.8 | Malignant neoplasm: Overlapping lesion of rectum anus and anal canal |
| C26.0 | Malignant neoplasm: Intestinal tract part unspecified |
| C26.8 | Malignant neoplasm: Overlapping lesion of digestive system |
| C78.5 | Secondary malignant neoplasm of large intestine and rectum |

*ICD-10, International Classification of Diseases, tenth revision; NEC, necrotizing enterocolitis; OPCS-4; Office of Population, Censuses and Surveys Classification of Surgical Operations and Procedures, fourth revision.*

**Supplementary** **Table 2: OPCS-4 codes for markers of anastomotic leakage**

| Procedure codes (OPCS-4) | | | |
| --- | --- | --- | --- |
| Code* | Description | Major AL | Minor AL |
| H113 | Colectomy and anastomosis NEC | Yes | No |
| H116 | Colectomy and end to side anastomosis NEC | Yes | No |
| H153 | Refashioning of colostomy | Yes | No |
| H161 | Drainage of colon | Yes | No |
| H178 | Other specified intra-abdominal manipulation of colon | Yes | No |
| H179 | Unspecified intra-abdominal manipulation of colon | Yes | No |
| H305 | Irrigation of colon | Yes | No |
| H308 | Other specified other operations on colon | Yes | No |
| H309 | Unspecified other operations on colon | Yes | No |
| H318 | Other specified image guided colorectal therapeutic operations | Yes | No |
| H319 | Unspecified image guided colorectal therapeutic operations | Yes | No |
| H321 | Resiting of colostomy | Yes | No |
| H334 | Anterior resection of rectum and anastomosis NEC | Yes | No |
| H331 | Abdominoperineal excision of rectum and end colostomy | Yes | No |
| H336 | Anterior resection of rectum and exteriorisation of bowel | Yes | No |
| H581 | Drainage of ischiorectal abscess | No | Yes |
| H588 | Other specified drainage through perineal region | No | Yes |
| H589 | Unspecified drainage through perineal region | No | Yes |
|  | | | |
| **Diagnosis codes (ICD-10)** | | | |
| **Code*** | **Description** | **Major AL** | **Minor AL** |
| K630 | Abscess of intestine | No | Yes |
| K650 | Acute Peritonitis | No | Yes |
| K658 | Other peritonitis | No | Yes |
| K659 | Peritonitis, unspecified | No | Yes |
| K918 | Other postprocedural disorders of digestive system, not elsewhere classified | No | Yes |
| K919 | Postprocedural disorder of digestive system, unspecified | No | Yes |
| T818 | Other complications of procedures, not elsewhere classified | No | Yes |
| Y832 | Surgical operation with anastomosis, bypass or graft | No | Yes |

**^*^**All codes denote AL. Those that indicate major/minor AL are marked as Yes.

*AL, anastomotic leak; ICD-10, International Classification of Diseases, tenth revision; NEC, necrotizing enterocolitis; OPCS-4; Office of Population, Censuses and Surveys Classification of Surgical Operations and Procedures, fourth revision.*

**Supplementary** **Table 3: All-cause inpatient costs within 90 days of follow-up and surgery hospitalisation costs (between 1 January 2018–31 December 2021) for matched patients with AL and without AL in GBP**

|  | **HCRU absolute values*** | | | | | | **HCRU statistical modelling* ^†^** | | | | |
| --- | --- | --- | --- | --- | --- | --- | --- | --- | --- | --- | --- |
| **Resource use metric** | **Overall AL cases** | | **Major AL cases** | | **Minor AL cases** | | **Overall AL cases** | | **Major AL cases** | | **Minor AL cases** |
|  | **With AL (n=1,982)** | **Without AL (n=1,982)** | **With AL (n=1,116)** | **With AL (n=1,982)  vs  without AL  (n=1,982)** | **With AL (n=866)** | **Without AL (n=866)** | **With AL (n=1,982)  vs  without AL  (n=1,982)** | | **With AL (n=1,116) vs  without AL  (n=1,1982)** | | **With AL  (n=866) vs  without AL (n=1,982)** |
| **Total all-cause cost including index surgery hospitalisation, ICU and subsequent hospital admission (£) within 90 days of follow-up** | | | | | | | | | | | |
| Mean (SD) | £23,878  (£32,193) | £12,070  (£13,043) | £26,474  (£39,137) | £11,488  (£12,244) | £20,564  (£19,627) | £12,821  (13,977) | **Adjusted difference (CI)** | | | | |
|  |  |  |  |  |  |  | £11,498:  (£10,111–£12,950)** | £14,194:  (£12,355–£16,182) | | £7,908:  (£6,277–£9,680) | |
| Median (IQR) | £16,411 (£10,539–£26,454) | £8,238 (£6,608–£12,707) | £18,001 (£11,367–£28,365) | £8,053 (£6,577–£12,303) | £14,387 (£9,689–£23,525) | £8,431 (£6,718–£13,069) |  | | | | |
| **Index surgery hospitalisation costs (£) including ICU episodes)** | | | | | | | | | | | |
| Mean (SD) | £19,802 (£30,910) | £11,019 (£11,846) | £21,666 (£37,842) | £10,467 (£10,756) | £17,405 (£18,259) | £11,734 (£13,094) |  | | | | |
| Median (IQR) | £11,844 (£7,397–£21,793) | £7,618 (£6,487–£11,243) | £12,172 (£7,120–£23,577) | £7,503 (£6,416–£10,920) | £11,559 (£7,858–£20,095) | £7,855 (£6,580–£11,832) |  |  |  |  |  |
| Subsequent admission costs (£ including ICU episodes) | | | | | | | | | | | |
| Mean (SD) | £3,955 (£9,917) | £1,028 (£4,531) | £4,762 (£11,704) | £1,038 (£5,298) | £2,921 (£6,858) | £1,015 (£3,297) |  | | | | |
| Median (IQR) | £634 (£0–£4,123) | £0 (£0–£490) | £786 (£0–£5,650) | £0 (£0–£471) | £411 (£0–£3,054) | £0 (£0–£512) |  |  |  |  |  |

**Absolute values are calculated based on matched AL cases compared with matched controls for AL cases and matched major/minor AL cases vs matched controls for major/minor cases; modelling data is calculated on the matched overall/major/minor AL cases vs the matched AL control cases only***^†^***All models have been adjusted for Charlson Comorbidity Index (CCI) and geographical region
**Indicates statistical significance (p<0.05)
AL, anastomotic leak; IQR, interquartile range; SD, standard deviation*

**References**

1. Zarnescu EC, Zarnescu NO, Costea R. Updates of Risk Factors for Anastomotic Leakage after Colorectal surgery. *Diagnostics (Basel)* 2021;**11**(12).

2. Ellis CT, Maykel JA. Defining Anastomotic Leak and the Clinical Relevance of Leaks. *Clin Colon Rectal Surg* 2021;**34**(6): 359-365.

3. Rahbari NN, Weitz J, Hohenberger W, Heald RJ, Moran B, Ulrich A, Holm T, Wong WD, Tiret E, Moriya Y, Laurberg S, den Dulk M, van de Velde C, Buchler MW. Definition and grading of anastomotic leakage following anterior resection of the rectum: a proposal by the International Study Group of Rectal Cancer. *Surgery* 2010;**147**(3): 339-351.

4. Spinelli A, Anania G, Arezzo A, Berti S, Bianco F, Bianchi PP, De Giuli M, De Nardi P, de Paolis P, Foppa C, Guerrieri M, Marini P, Persiani R, Piazza D, Poggioli G, Pucciarelli S, D'Ugo D, Renzi A, Selvaggi F, Silecchia G, Montorsi M. Italian multi-society modified Delphi consensus on the definition and management of anastomotic leakage in colorectal surgery. *Updates Surg* 2020;**72**(3): 781-792.

5. van Helsdingen CP, Jongen AC, de Jonge WJ, Bouvy ND, Derikx JP. Consensus on the definition of colorectal anastomotic leakage: A modified Delphi study. *World J Gastroenterol* 2020;**26**(23): 3293-3303.

6. Daniel VTA, K.; Davids, J.S.; Sturrock, P.R.; Harnsberger, C.R.; Steele, S.R.; Maykel, J.A. . The utility of the delphi method in defining anastomotic leak following colorectal surgery.*Am J Surg* 2020;**219**(1): 75-79.

7. Heuvelings DJI, Bouvy ND, Francis N, van Kuijk SMJ, Kimman ML, Boutros M, Sylla P, CoRe ALc. International Consensus on Reporting Anastomotic Leaks After Colorectal Cancer Surgery: The CoReAL Reporting Framework. *Dis Colon Rectum* 2025.

8. European Society of Coloproctology Collaborating G. The 2017 European Society of Coloproctology (ESCP) international snapshot audit of left colon, sigmoid and rectal resections - Executive Summary. *Colorectal Dis* 2018;**20 Suppl 6**: 13-14.

9. McArdle COLORECTAL SURGERY, McMillan DC, Hole DJ. Impact of anastomotic leakage on long-term survival of patients undergoing curative resection for colorectal cancer. *Br J Surg* 2005;**92**(9): 1150-1154.

10. McDermott FD, Heeney A, Kelly ME, Steele RJ, Carlson GL, Winter DC. Systematic review of preoperative, intraoperative and postoperative risk factors for colorectal anastomotic leaks. *Br J Surg* 2015;**102**(5): 462-479.

11. Hammond J, Lim S, Wan Y, Gao X, Patkar A. The burden of gastrointestinal anastomotic leaks: an evaluation of clinical and economic outcomes. *J Gastrointest Surg* 2014;**18**(6): 1176-1185.

12. Kube R, Mroczkowski P, Granowski D, Benedix F, Sahm M, Schmidt U, Gastinger I, Lippert H, Study group Qualitatssicherung KR-K. Anastomotic leakage after colon cancer surgery: a predictor of significant morbidity and hospital mortality, and diminished tumour-free survival. *Eur J Surg Oncol* 2010;**36**(2): 120-124.

13. Krarup PM, Nordholm-Carstensen A, Jorgensen LN, Harling H. Association of Comorbidity with Anastomotic Leak, 30-day Mortality, and Length of Stay in Elective Surgery for Colonic Cancer: A Nationwide Cohort Study. *Dis Colon Rectum* 2015;**58**(7): 668-676.

14. Bertelsen CA, Andreasen AH, Jorgensen T, Harling H, Danish Colorectal Cancer G. Anastomotic leakage after curative anterior resection for rectal cancer: short and long-term outcome. *Colorectal Dis* 2010;**12**(7 Online): e76-81.

15. Lee SW, Gregory D, Cool CL. Clinical and economic burden of colorectal and bariatric anastomotic leaks. *Surg Endosc* 2020;**34**(10): 4374-4381.

16. Enodien B, Maurer A, Ochs V, Bachmann M, Gripp M, Frey DM, Taha A. The Effects of Anastomotic Leaks on the Net Revenue from Colon Surgery. *Int J Environ Res Public Health* 2022;**19**(15).

17. Flor-Lorente B, Noguera-Aguilar JF, Delgado-Rivilla S, Garcia-Gonzalez JM, Rodriguez-Martin M, Salinas-Ortega L, Casado MA, Alvarez M. The economic impact of anastomotic leak after colorectal cancer surgery. *Health Econ Rev* 2023;**13**(1): 12.

18. Ashraf SQ, Burns EM, Jani A, Altman S, Young JD, Cunningham C, Faiz O, Mortensen NJ. The economic impact of anastomotic leakage after anterior resections in English NHS hospitals: are we adequately remunerating them? *Colorectal Dis* 2013;**15**(4): e190-198.

19. La Regina D, Di Giuseppe M, Lucchelli M, Saporito A, Boni L, Efthymiou C, Cafarotti S, Marengo M, Mongelli F. Financial Impact of Anastomotic Leakage in Colorectal surgery. *J Gastrointest Surg* 2019;**23**(3): 580-586.

20. Capolupo GT, Galvain T, Parago V, Tong C, Masciana G, Di Berardino S, Caputo D, La Vaccara V, Caricato M. In-hospital economic burden of anastomotic leakage after colorectal anastomosis surgery: a real-world cost analysis in Italy. *Expert Rev Pharmacoecon Outcomes Res* 2022;**22**(4): 691-697.

21. Nijssen DJ, Wienholts K, Postma MJ, Tuynman J, Bemelman WA, Laméris W, Hompes R. The economic impact of anastomotic leakage after colorectal surgery: a systematic review. *Tech Coloproctol* 2024;**28**(1): 55.

22. Zoucas E, Lydrup M-L. Hospital costs associated with surgical morbidity after elective colorectal procedures: a retrospective observational cohort study in 530 patients. *Patient Saf Surg* 2014;**8**(1): 2.

23. https://ec.europa.eu/eurostat/databrowser/view/tec00114__custom_19267291/default/ table. Purchasing power parity for GDP per capita for UK compared to EU 27 (100) obtained for years 2022 (1.02) and 2013 (1.10), last updated 10 July 2025. Accessed 09 Dec 2025.

24. Bischofberger S, Adshead F, Moore K, Kocaman M, Casali G, Tong C, Roy S, Collins M, Brunner W. Assessing the environmental impact of an anastomotic leak care pathway. *Surg Open Sci* 2023;**14**: 81-86.

25. Hospital Episode Statistics (HES). <https://digital.nhs.uk/services/hospital-episode-statistics> [11 February 2025].

26. Gupta R, Gupta J, Ammar H. Impact of COVID-19 on the outcomes of gastrointestinal surgery. *Clin J Gastroenterol* 2021;**14**(4): 932-946.

27. Burns EM, Bottle A, Aylin P, Darzi A, Nicholls RJ, Faiz O. Variation in reoperation after colorectal surgery in England as an indicator of surgical performance: retrospective analysis of Hospital Episode Statistics. *BMJ* 2011;**343**: d4836.
